# SIRT6 deficiency impairs the deacetylation and ubiquitination of UHRF1 to strengthen glycolysis and lactate secretion in bladder cancer

**DOI:** 10.1186/s13578-024-01333-2

**Published:** 2024-12-21

**Authors:** Xiaojing Wang, Peipei Zhang, Jiaqi Yan, Jingyi Huang, Yan Shen, Hongchao He, Hongjing Dou

**Affiliations:** 1 Department of Urology, Ruijin Hospital, Shanghai Jiao Tong University School of Medicine, Shanghai, 200025 China; 2 The State Key Laboratory of Metal Matrix Composites, School of Materials Science and Engineering, Shanghai Jiao Tong University, Shanghai, 200240 China; 3 Research Centre for Experimental Medicine, Ruijin Hospital, Shanghai Jiao Tong University School of Medicine, Shanghai, 200025 China

**Keywords:** SIRT6, UHRF1, Deacylation, Lactate, BLCA

## Abstract

**Background:**

Aberrant interplay between epigenetic reprogramming and metabolic rewiring events contributes to bladder cancer progression and metastasis. How the deacetylase Sirtuin-6 (SIRT6) regulates glycolysis and lactate secretion in bladder cancer remains poorly defined. We thus aimed to study the biological functions of SIRT6 in bladder cancer.

**Methods:**

Bioinformatic analysis was used to study the prognostic significance of SIRT6/UHRF1 in BLCA. Both in vitro and in vivo assays were used to determine the roles of SIRT6/UHRF1 in BLCA. Deacetylation and ubiquitin assays were performed to uncover the regulations of SIRT6-UHRF1. Measurement of extracellular acidification rate (ECAR) and oxygen consumption rate (OCR) was used to assess glycolytic abilities.

**Results:**

Here, we show that protein deacetylase SIRT6 was down-regulated in BLCA, and predicts poor overall survival. SIRT6 deficiency notably enhances BLCA cell proliferation, self-renewal, and migration capacities in vitro and in vivo. Mechanistically, SIRT6 interacts with, deacetylates, and promotes UHRF1 degradation mediated by β-TrCP1. Thus, SIRT6 deficiency leads to stabilized UHRF1 and depends on UHRF1 to accelerate BLCA malignant progression. Furthermore, UHRF1 significantly increased aerobic glycolysis via activating MCT4/HK2 expressions. Down-regulated SIRT6 thus depended on UHRF1 to promote glycolysis and lactate secretion in BLCA. Targeting UHRF1 or MCT4 notably impaired the extracellular lactate accumulations in BLCA. Significantly, a specific small-molecule inhibitor (NSC232003) targeting UHRF1 substantially inhibited SIRT6-deficient BLCA progression.

**Conclusion:**

Together, our study uncovered an epigenetic mechanism of the SIRT6/UHRF1 axis in driving BLCA glycolysis and lactate secretion, creating a novel vulnerability for BLCA treatment.

**Supplementary Information:**

The online version contains supplementary material available at 10.1186/s13578-024-01333-2.

## Introduction

Bladder urothelial carcinoma (BLCA) is currently the most common malignant tumor in the urinary system, and the incidence of bladder cancer has increased over the years [1]. The American Cancer Society estimates that the numbers of new BLCA cases and deaths in the United States of 2024 were 81,180 and 17,100, respectively [2]. Recently, surgical procedures have become the standard therapeutic intervention for localized and non-muscle-invasive BLCA (NMIBC) patients [3]. However, nearly 20% of NMIBC cases would progress to muscle-invasive bladder cancer (MIBC) that may spread to tissues and organs outside the bladder or metastasize to lymph nodes. MIBC cases are mainly treated with surgery, radiotherapy, chemotherapy, etc. [4]. However, the above strategies are often limited in advanced MIBC, owing to its persistently high recurrence rates. As a result, it has become an essential issue to identify effective biological targets for treating BLCA.

Without altering the DNA sequences, epigenetics is the study of heritable modifications of DNA or associated proteins that modulate gene expression during cell division, growth, or evolution [5]. So far, the epigenetic mechanisms mainly include four groups: DNA methylation, histone post-translational modifications or chromatin remodeling, histone variants, and non-coding RNAs’ regulation [6]. Several well-known enzymes are reported to contribute to these epigenetic modifications, including DNA methyltransferases (DNMTs) and demethylases (TETs), histone methyltransferases (HTMs), and demethylases (HDMs), as well as histone acetyltransferases (HATs) and deacetylases (HDACs)[7, 8]. As is well reported, the reversible functions of histone acetyltransferases (HATs) and histone deacetylases (HDACs) contribute to the “acetyl-chromatin” homeostasis [9]. The HDAC/SIRT family members could be further categorized into five groups, some differentially expressed in normal and urinary bladder tissues. The silent information regulator 2 protein (sirtuin) family is conserved deacetylase and depends on nicotinamide adenine dinucleotide (NAD^+^) [10]. As a NAD^+^-dependent histone deacetylase, SIRT6 is a repressor of MYC-driven transcription and participates in glucose metabolism, inflammatory homeostasis, and longevity [11]. Mainly localized in the nucleus, SIRT6 is responsible for deacetylation of histone H3 N-acetyl-lysine 9 (H3K9ac) and histone H3 N-acetyl-lysine 56 (H3K56ac) to modulate the gene transcription [12]. Previous studies have implicated that SIRT6 may serve as a tumor suppressor. For instance, MOF-mediated SIRT6 acetylation hinders the interaction between SIRT6 and transcriptional factor FOXA2, which in turn leads to the transcriptional activation of ZEB2, thus promoting NSCLC progression [13]. In addition, the abnormal METTL14-USP48 axis contributes to SIRT6 stabilization that suppresses liver cancer glycolysis and malignancy [14]. However, SIRT6 could also have pro-tumorigenic functions. For instance, SIRT6 represses the expressions of the T-box transcription factor 3 (Tbx3) by deacetylation of H3K9ac, which is predictive of poor prognosis in HER2-positive breast cancer patients [15]. Besides, increased proline synthesis promotes deacetylases SIRT6/7-mediated deacetylation of histone H3 at lysine 27 (H3K27) and thereby biases a global transcriptional output toward a neuroendocrine lineage profile in prostate and lung cancers [16]. Despite the importance of SIRT6 in multiple tumors, the molecular basis of the regulatory process that SIRT6 drives in BLCA remains little understood.

Metabolic switching toward glycolysis is a hallmark of cancer, by which tumors enhance energy utilization, distant metastasis, and immune evasion [17]. Glycolysis is defined as the process by which one molecule of glucose is decomposed into two molecules of pyruvate, along with two molecules of ATP [18]. In an aerobic condition, pyruvate usually enters mitochondria for the tricarboxylic acid cycle (TAC) and generates a large amount of ATP. Nevertheless, when the cell is in hypoxia or lack of mitochondria, pyruvate is reduced to lactate. As one of the products of glycolysis, lactate plays an essential role in cancer progression, like tumor growth, angiogenesis, metastasis, as well as immune escape [19]. For instance, Zheng Cao et al. found that lactate oxidase nanocapsules potentiate T cell immunity and efficacy of cancer immunotherapy [17]. CDK7-YAP-LDHD crosstalk enhances D-lactate elimination and ferroptosis resistance to sustain tumor stem cell-like capacities in esophageal squamous cell carcinoma (ESCC) [20]. In bladder cancer, Circ_0000235 activates MCT4 to promote glycolysis and tumor proliferation by sponging miR-330-5p, implicating that MCT4-dependent lactate metabolism contributes to BLCA progression [21]. However, the underlying molecular mechanisms by which these upstream crosstalk activate the lactate metabolism pathway remain unclear.

Here, we revealed a novel mechanism for SIRT6 in driving BLCA and identified UHRF1 (Ubiquitin-like, containing PHD and RING Finger domains 1), an essential epigenetic translational factor, as a new SIRT6 downstream substrate. We showed that SIRT6 deacetylates UHRF1 and triggers β-TrCP1-mediated UHRF1 degradation. SIRT6 deficiency thus leads to UHRF1 stabilization and drives the UHRF1/MCT4 axis to enhance lactate release in BLCA. Our present study may show a novel axis of SIRT6/UHRF1/MCT4 that links abnormal epigenetic events and metabolic rewiring in BLCA.

## Methods and materials

### Cell culture and transfection

SIRT6^high^ (T24, UMUC-3, BIU-87) and SIRT6^low^ (5637, HT-1376, 253J) BLCA cells were obtained from either the American Type Culture Collection (ATCC). The identification of the cell lines was verified by short tandem repeat (STR) genotyping. The cell lines were cultivated in conventional RPMI 1640 (T24, UMUC-3, HT-1376) or DMEM (BIU87, 5637, 253J, 293 T) with the addition of 10% fetal bovine serum (FBS).

### Quantitative real-time PCR

TRIzol solution (Invitrogen, Carlsbad, CA, USA) was utilized to get total RNA from cells or tissues. Reverse transcription (RT) was conducted with HiScript Q RT SuperMix for qPCR (Vazyme, Jiangsu, China). RT-PCR was performed in triplicate reactions via an Applied Biosystems 7900HT sequence detection system and a SYBR Green PCR Kit (Vazyme, Jiangsu, China). The following is a list of primers that were utilized (5'- > 3'): SIRT6: CCCACGGAGTCTGGACCAT (Forward), CTCTGCCAGTTTGTCCCTG (Reverse); UHRF1: AGGTCAATGAGTACGTCGATGC (Forward), TTCTCCGGGTAGTCGTCGT (Reverse); MCT4: CCATGCTCTACGGGACAGG (Forward), GCTTGCTGAAGTAGCGGTT (Reverse); HK2: GAGCCACCACTCACCCTACT (Forward), CCAGGCATTCGGCAATGTG (Reverse).

### Cell transfection and RNA interference

For the lentiviral plasmids, 293 T cells were utilized for virus production. Briefly, 80% confluency of 293 T cells was applied to transfect with helper plasmids psPAX2/pMD2.G and lentiviral vector plasmids in 6-well plates. After incubation overnight, cells were replaced with 1.5 ml fresh medium, and the virus was collected after one day; cells were changed with the fresh medium again for a 2nd collection after another day of culturing. 0.45 mm filters were used to filter the virus. BLCA cell lines were seeded in 6-well plates to be 30–50% confluent at infection, 200 ml virus/well was applied, and 6–8 mg/mL polybrene was included to increase efficiency during the infection. Media were changed after 12–24 h infection, and the cells underwent appropriate antibiotic selection after more than 24 h incubation. The oligonucleotide sequences were designed and listed as the following (5'- > 3'): shSIRT6: CCGGTGGAAGAATGTGCCAAGTGTACTCGAGTACACTTGGCACATTCTTCCATTTTTG (Forward), AATTCAAAAATGGAAGAATGTGCCAAGTGTACTCGAGTACACTTGGCACATTCTTCCA (Reverse); shUHRF1: CCGGTGTGAAATACTGGCCCGAGAACTCGAGTTCTCGGGCCAGTATTTCACATTTTTG (Forward), AATTCAAAAATGTGAAATACTGGCCCGAGAACTCGAGTTCTCGGGCCAGTATTTCACA (Reverse); shMCT4: CCGGGCTCATACAGGAGTTTGGGATCTCGAGATCCCAAACTCCTGTATGAGCTTTTTG (Forward), AATTCAAAAAGCTCATACAGGAGTTTGGGATCTCGAGATCCCAAACTCCTGTATGAGC (Reverse); shCtrl: CCGGCAACAAGATGAAGAGCACCAACTCGAGTTGGTGCTCTTCATCTTGTTGTTTTTG (Forward), AATTCAAAAACAACAAGATGAAGAGCACCAACTCGAGTTGGTGCTCTTCATCTTGTTG (Reverse).

### CCK-8 and colony formation assay

In the CCK-8 experiments, the tumor cells were placed in a 96-well plate at a density of 3,000 cells per well, with each well containing 100 µl of DMEM supplemented with 10% FBS. The original medium of each group was changed on separate days with 10 µl of CCK-8 solution diluted in 100 µl of complete culture medium, by the CCK-8 solution protocol (Dojindo, Kumamoto, Japan). Following a 2-h incubation period at 37 °C in the absence of light, we identified live cells by measuring their absorbance at 450 nm wavelength. BLCA cells stably expressed shRNA against SIRT6 or UHRF1, and the relative control cells were seeded. After cultivating for 10 days, 4% paraformaldehyde was used to fix the cells, followed by staining with 1% crystal violet. The colonies were counted subsequently.

### Assays for migration and invasion

The migratory and invasive characteristics of tumor cells were evaluated via transwell assays performed in 24-well transwell plates (Corning, NY, USA). In migration tests, we introduced 40,000 BLCA cells into the top chamber with 200 µl of DMEM without serum and subsequently added 500 µl of DMEM with 30% FBS to the bottom chamber. To conduct the invasion tests, we applied a 1:6 combination of Matrigel (BD Biosciences) and DMEM to the chamber inserts, coating them with 50 µl, and the inserts were then incubated at 37 °C for 2 h. Subsequently, we introduced 80,000 cells into the top chamber. The bottom chamber contained 500 µl of DMEM with 30% FBS. Following a 48-h incubation period, we used a 4% paraformaldehyde solution to immobilize the cells that had migrated or invaded the bottom of the membrane. Then, the fixed cells were stained with crystal violet for 15 min. 5 arbitrary 100 × microscopic areas were chosen to enumerate the labeled cells using an IX71 inverted microscope manufactured by Olympus Corporation. We conducted the research by performing all our tests three times to ensure accuracy and reliability.

### In vitro deacetylation assay

Briefly, SIRT6-KD BLCA cells were pre-treated with HDAC inhibitors (10 mM NAM, 50 nM TSA, 5 mM Sodium butyrate) for 6 h, then lysed in 300 mM KCl, 20 mM Tris–HCl (pH 7.9), 5 mM MgCl2, 0.2 mM EDTA, 10% glycerol, 0.5 mM DTT supplemented with 0.1% NP-40, protease inhibitors (Roche) and HDAC inhibitors (Sigma). UHRF1 was purified and enriched. The acetylation level of UHRF1 was monitored by western blotting with anti-pan-acetylation lysine antibodies.

### Ubiquitin assay

HEK293T cells were transfected with an HA-ubiquitin construct together with indicated plasmids. After 48 h, cells were harvested in the immunoprecipitation buffer mentioned above by sonication. The ubiquitination of indicated proteins was immunoprecipitated and analyzed by western blotting with an anti-ubiquitin antibody.

### Western blotting and immunoprecipitation

For western blotting, the protein extracts were produced using Laemmli loading buffer. Subsequently, they were separated using SDS polyacrylamide gels and deposited onto a PVDF membrane (Millipore). Finally, the membranes were probed with the corresponding antibodies, and the Bio-Rad system was used to visualize the immunoblots. To facilitate immunoprecipitation, the indicated cells that received the specified treatments were broken down in a solution containing 200 mM KCl, 20 mM Tris–HCl (pH 7.9), 5 mM MgCl_2_, 10% glycerol, 0.2 mM EDTA, and 0.1% NP-40. This solution was further treated with protease inhibitors from Roche Complete. Subsequently, the lysates of clear cells were treated overnight at 4 °C with the appropriate antibodies or control IgGs. Immunoprecipitates bound to beads were washed, rinsed in Laemmli loading buffer, and subjected to western blotting analysis. The following antibodies were utilized in our study: anti-SIRT6 (Abcam, ab289970), anti-UHRF1 (Abcam, ab213223), anti-MCT4 (Abcam, ab308528), anti-Hexokinase II (Cell Signaling Technology, CST#2867), anti-GAPDH (Abcam, ab8245).

### Immunohistochemical (IHC) staining

For IHC experiments, 4μm formalin-fixed, paraffin-embedded (FFPE) tissue sections were deparaffinized with xylene and rehydrated with graded alcohol incubations. For SIRT6 (Abcam, ab289970), UHRF1(Abcam, ab213223) staining, pressurized antigen retrieval was performed at 125 °C for 4min (BioGenex, HK080-9K), followed by a 30 min depressurization period and an additional 30 min room temperature cooling period. Sections were treated with 3% H_2_O_2_ for 10min, permeabilized with 0.1% Tween-20 for 20 min, and blocked with 5% goat serum/0.3% Triton X-100 in phosphate-buffered saline (PBS) for 1h before incubation with primary antibody overnight at 4 °C. Subsequent incubations were performed as described with MYC staining; however, all washes were done with either PBS or PBS with 0.1% Tween-20. Whole slide bright field imaging was performed with the Zeiss Axio Scan.Z1 microscope (20 × objective lens).

### Measurement of extracellular acidification rate (ECAR) and oxygen consumption rate (OCR)

Cellular mitochondrial function and glycolytic capacity were measured using the Seahorse Bioscience XF96 Extracellular Flux Analyzer, according to the manufacturer's instructions of Seahorse XF Cell Mito Stress Test Kit or Glycolysis Stress Test Kit (Seahorse Bioscience, Billerica, MA, USA). Cells were plated in XF96 Cell Culture Microplates (Seahorse Bioscience) at an initial cellular density of 4 × 10^4^ cells/well the day before determination. Seahorse buffer consists of DMEM, phenol red, 25 mM glucose, 2 mM sodium pyruvate, and 2 mM glutamine. For ECAR measurement, 10 mM glucose, 1 μM oligomycin, and 100 mM 2-deoxy-glucose were automatically added to measure ECAR value. After monitoring baseline respiration, 1 μM oligomycin, 1 μM FCCP(Carbonyl cyanide-4-(trifluoromethoxy)phenylhydrazone), and 1 μM rotenone was automatically injected into XF96 Cell Culture Microplates to measure OCR. ECAR and OCR values were calculated after normalization to cell number.

### Lactate/glucose detection

Cells were plated into 6-wells plate at a density of 2 × 105 cells/well for culturing 24h. Both cell lysis and medium were collected. For cell lysis, resuspend cell in 100ul assay buffer and sonicated for 3min (10s on, 12s off, 240W). Then centrifuge and collect the supernatant. Lactate detection was performed using Assay kits (ScienCell, Carlsbad, CA, USA, #8308) following the manufacturer’s instruction and then normalized to total protein.

### Xenograft models and experiments

The BALB/c nude mice that were devoid of pathogens were acquired from the Slaccas in Shanghai. The housing and handling of all mice were conducted by the authorized procedures of the Ethics Committee of Ruijin Hospital, Shanghai Jiaotong University. We conducted preliminary studies to ascertain the requisite sample size of mice, and the assignment of mice to experimental groups was conducted randomly. The calculation of tumor volume was performed using the following formula:

$${\text{Volume }}\left( {{\text{mm}}^{{3}} } \right)\, = \,0.{52}\, \times \,{\text{length }}\left( {{\text{mm}}} \right)\, \times \,{\text{width }}\left( {{\text{mm}}} \right)^{{2}} .$$

### Statistical analysis

Each experiment was conducted with a minimum of three replicates. All data were presented as mean ± S.D. or mean ± S.E.M., as mentioned in the figure captions. To compare the central tendency of normally distributed data, unpaired two-sided Student's t-tests were conducted assuming equal variance. Non-parametric Mann–Whitney U tests were used to analyze data sets that were not normally distributed. Statistical significance was determined for differences when the P-value was less than 0.05.

## Results

### SIRT6 is down-regulated in BLCA and correlates with a poor prognosis in BLCA patients

**Fig. 1:**
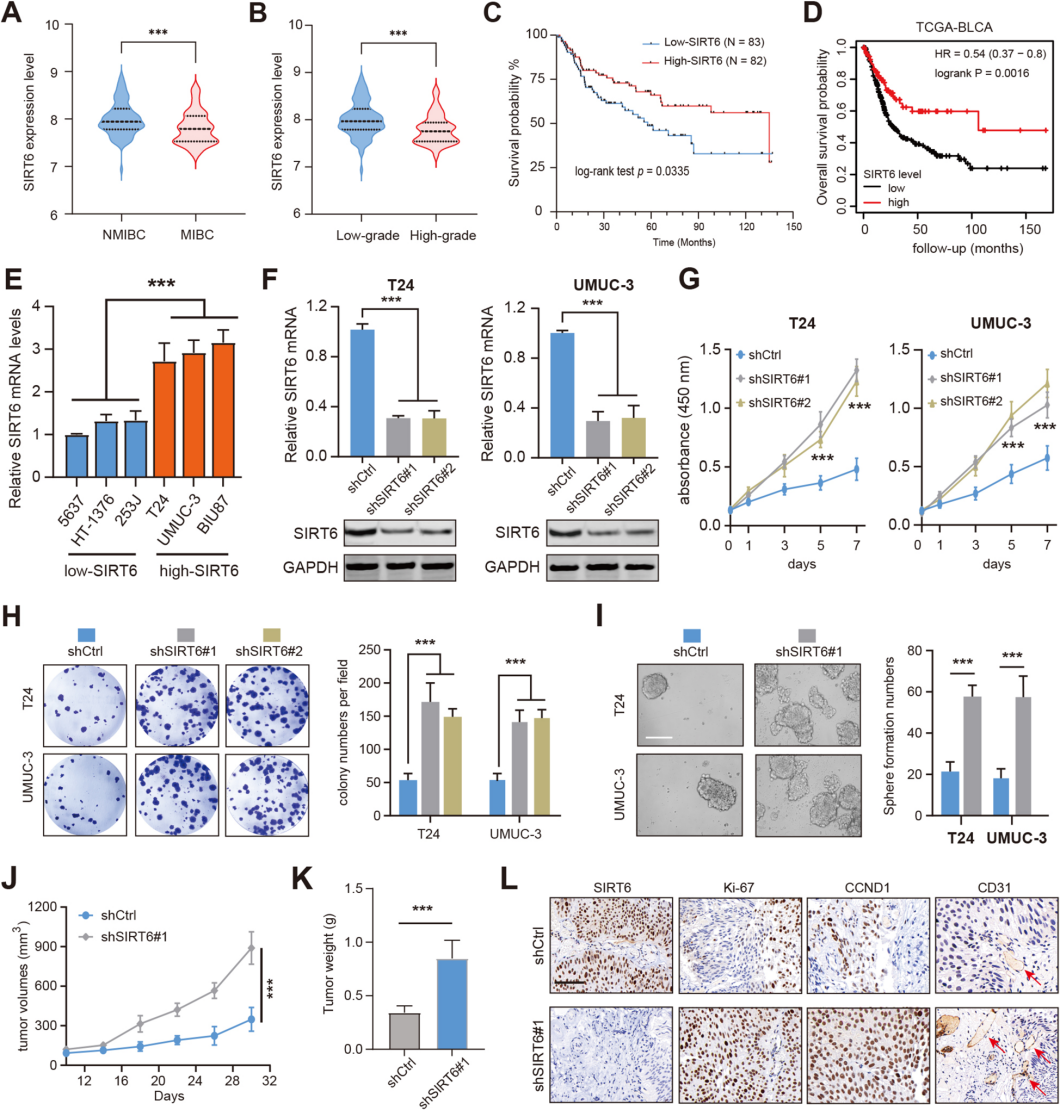
The prognostic analysis of SIRT6 in BLCA and its functional validations.** A** Differential analysis of SIRT6 expression levels between NMIBC and MIBC samples.** B** Differential analysis of SIRT6 expression levels between high-grade and low-grade BLCA samples. **C** Kaplan–Meier analysis shows the differential prognosis between SIRT6-high and SIRT6-low BLCA patients in GSE13507. **D** Kaplan–Meier analysis shows the differential prognosis between SIRT6-high and SIRT6-low BLCA patients in TCGA-BLCA. **E** RT-qPCR analysis was conducted to determine SIRT6-high and SIRT6-low BLCA cell lines. **F** Western blotting assays showing the SIRT6 proteins in T24 and UMUC-3 cells with or without SIRT6 depletion. **G** Knockdown of SIRT6 increased cell viability as indicated by CCK-8 proliferation assay. **H** SIRT6 knockdown in T24 or UMUC-3 cells increased the colony formation capacity of these cells. **I** Sphere formation assays showing the self-renewal capacities of control or shSIRT6 BLCA cells. **J-K** Quantification of subcutaneous tumor growth curve (**J**) and weights (**K**) of tumors derived from control and SIRT6-KD UMUC-3 cells (2-way ANOVA followed by Tukey’s multiple comparisons tests); scale bar = 1 cm. **L** Representative IHC graphs showing the intensity of SIRT6, Ki-67, CCND1, and CD31 in tumors derived from control and SIRT6-KD UMUC-3 cells. *p < 0.05, **p < 0.01, ***p < 0.001, ns no significance

Previous research implicated that SIRT6 may exert opposite functions in multiple tumors, and serve as a tumor suppressor or an oncogene. Besides, it is still unknown whether SIRT6 is aberrantly expressed in BLCA that influences tumor progression. To clarify this point, we obtained the expression pattern of SIRT6 in BLCA patients using the publicly available GSE13507 dataset. We found that SIRT6 was down-regulated in muscle-invasive bladder cancer (MIBC) samples compared with non-muscle-invasive bladder cancer (NMIBC) tissues (Fig. 1A). Consistently, down-regulated SIRT6 levels were associated with higher tumor grades (Fig. 1B). Kaplan–Meier survival curve analysis indicated that low SIRT6 expression level was significantly correlated with shorter overall survival (OS) in the GSE13507 dataset (Fig. 1C). In another publicly accessible TCGA-BLCA cohort, low SIRT6 expression was also notably associated with worse OS in patients (Fig. 1D). Thus, the bioinformatic analysis suggested that SIRT6 may be a tumor suppressor in BLCA, and low levels of SIRT6 predict poor prognosis. To validate the statistical findings, a series of standard BLCA cell lines were collected and categorized into SIRT6-high or SIRT6-low groups based on the SIRT6 expression levels (Fig. 1E). We selected two SIRT6-high BLCA cell lines (T-24, UMUC-3) and generated two stable SIRT6-knockdown (KD) sublines by transfecting cells with two shRNAs targeting different regions of the SIRT6 mRNA. RT-qPCR and western blot showed lower SIRT6 mRNA and protein levels in these sublines, respectively (Fig. 1F). SIRT6 depletion dramatically elevated the proliferation rate of T-24 or UMUC-3 cells, as quantified by the total cell viability over one week (Fig. 1G). SIRT6 depletion further led to notably more and larger colonies by the colony formation assays (Fig. 1H). Interestingly, an increase in sphere numbers and sizes was found in SIRT6-depleted T-24 and UMUC-3 cells compared with the control cells (Fig. 1I). Lastly, we conducted the murine xenograft assay to examine the in vivo role of SIRT6 in BLCA. As expected, the tumor growth curve suggested that the UMUC-3 cells with SIRT6-KD formed larger tumors than the control cells (Fig. 1J-K). The immunohistochemistry (IHC) assay further detected that expressions of proliferation marker (Ki-67), and aginogenesis (CD31) were much higher in SIRT6-KD tumors than in control tumors (Fig. 1L). Taken together, these results from in vitro and in vivo models confirmed the suppressor role of SIRT6, and targeting SIRT6 enhanced BCLA growth and stemness.

### SIRT6 deficiency enhanced BLCA metastasis in vitro and in vivo

**Fig. 2:**
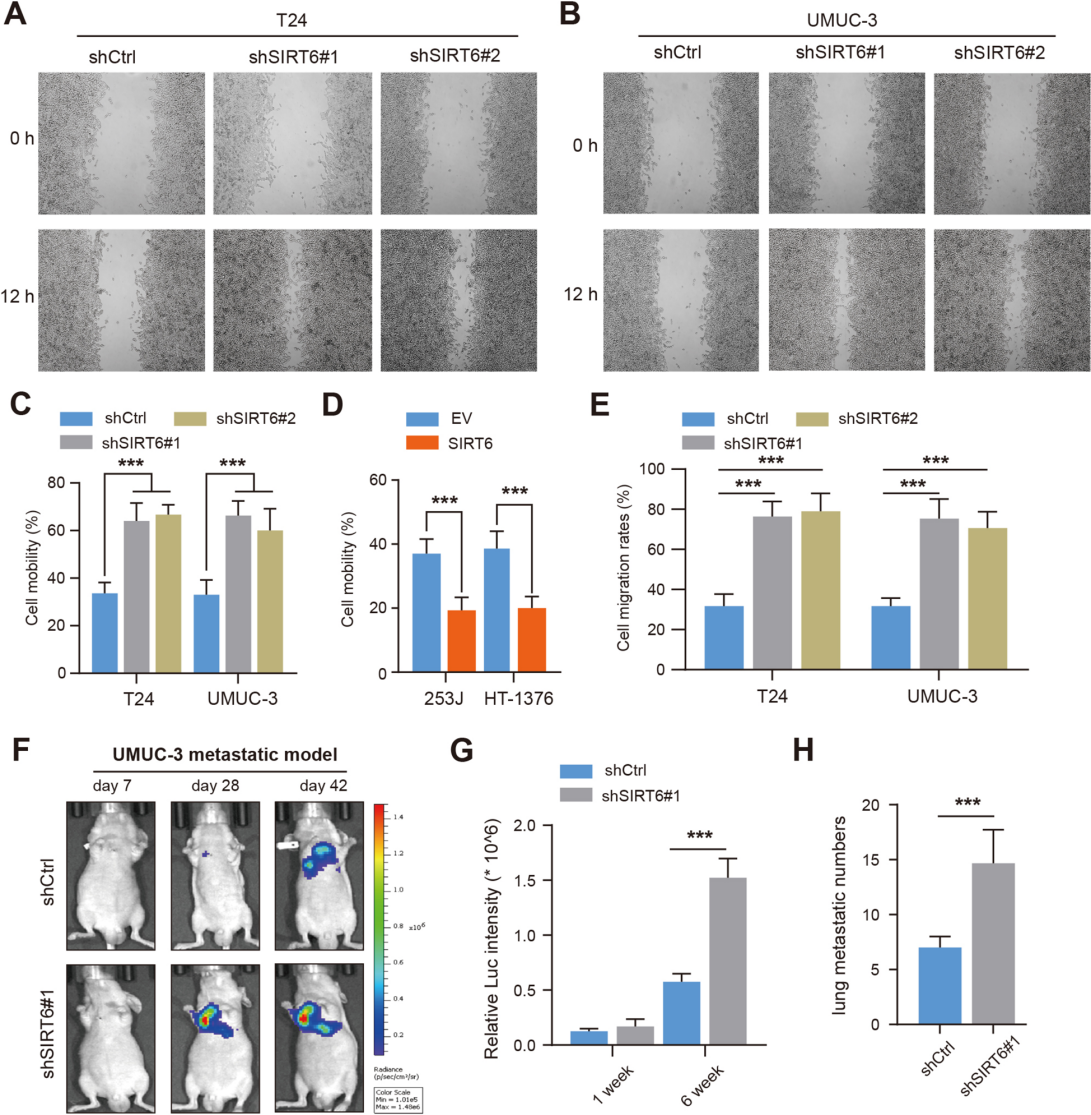
SIRT6 deficiency promoted BLCA cell metastasis abilities in vitro and in vivo. **A-C** Wound healing assay of SIRT6-KD T24 (A) and UMUC-3 cells (**B**) and quantification analysis (**C**). Scale bar, 100 µm. **D** Wound healing assay and quantification analysis in control and SIRT6-overexpressing 253J or HT-1376 cells. **E** Quantification of transwell assays for SIRT6-KD T24 and UMUC-3 cells. Cell number was counted in six randomly captured pictures. **F** Representative bioluminescence (BIL) pictures showing the metastatic lesions in mice injected with control or SIRT6-KD UMUC-3 cells individually. **G-H** The BIL signals and metastatic lung lesions were quantified, respectively. *p < 0.05, **p < 0.01, ***p < 0.001, ns no significance

We next performed several experiments to test the hypothesis that SIRT6 may also modulate the BLCA metastasis process. The wound-healing assays were performed accordingly, and we found that SIRT6 deficiency could lead to faster wound closure in T-24 and UMUC-3 cells (Fig. 2A-C). Compared with control cells, SIRT6 overexpression significantly impeded the migration efficiency in 253J and HT-1376 cells (Fig. 2D). In addition, transwell assays further confirmed that SIRT6 deficiency promoted the invasion abilities of T-24 and UMUC-3 cells, whereas SIRT6 overexpression attenuated the abilities (Fig. 2E, and Figure S1A-B). To further confirm the metastasis-promoting functions of SIRT6 in BLCA, we generated UMUC-3-luciferase cells transfected with NC or shSIRT6#1 and injected them into the tail veins of nude mice individually (Fig. 2F). After 6 weeks injection, we found higher metastatic signals and more microscopic metastatic nodules in the mice derived from shSIRT6 group than those in control group (Fig. 2G, H). Collectively, these findings suggested that SIRT6 deficiency significantly enhanced BLCA cell migration in vitro and in vivo.

### SIRT6 deacetylates UHRF1 and triggers β-TrCP1-mediated UHRF1 degradation

**Fig. 3:**
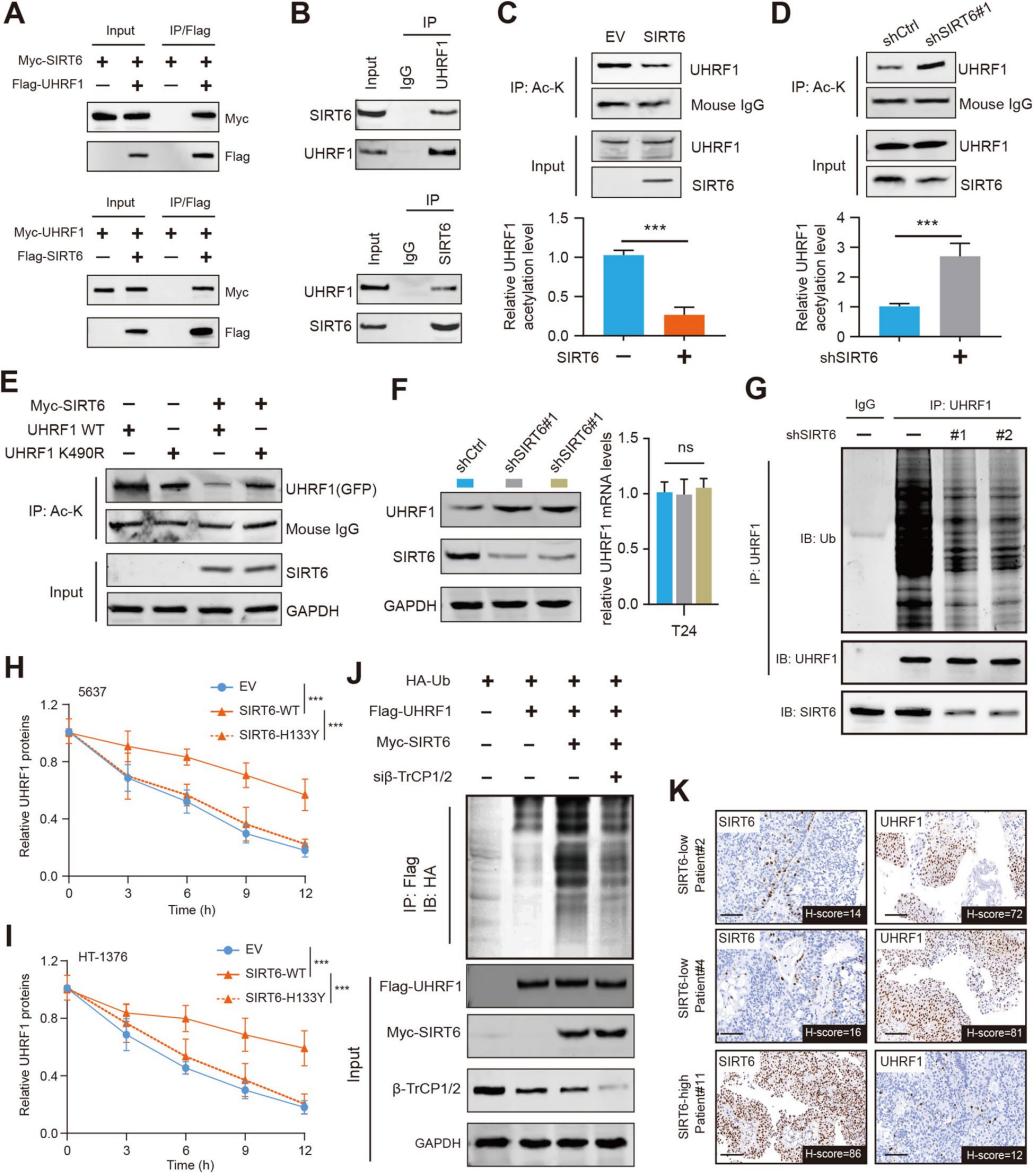
SIRT6 deacetylates UHRF1 and promotes β-TrCP1-mediated UHRF1 degradation. **A** Western blots of indicated proteins in WCL and co-IP samples of anti-FLAG antibody obtained from 293 T cells transfected with indicated plasmids. **B** The co-immunoprecipitation (co-IP) assay showing the endogenous interactions between SIRT6 and UHRF1 proteins. **C** Acetylation level of UHRF1 in 253J cells transfected with EV or SIRT6-OE plasmids probed with pan anti acetyl lysine antibodies. Lower is quantitative data showing a decreased level of UHRF1 acetylation in cells with SIRT6 OE. **D** Acetylation level of UHRF1 in T24 cells treated with shCtrl or shSIRT6 lentiviruses probed with pan anti acetyl lysine antibodies. Lower is quantitative data showing an increased level of UHRF1 acetylation in cells with SIRT6-KD. **E** 253J shUHRF1 cells overexpressing SIRT6 and UHRF1 WT or UHRF1 K490R were used to measure the acetylation level of UHRF1 at residue K490. **F** Western blotting and RT-qPCR assays detecting the UHRF1 expression levels in shCtrl and shSIRT6#1 BLCA cells. **G** Detection of UHRF1 ubiquitination by IP and IB as indicated in control and SIRT6-KD cells. **H-I** UHRF1 proteins in WCLs of 5637 (**H**) or HT-1376 cells (**I**) transfected with the indicated SIRT6 plasmids for 48 h and then treated with CHX (50 μg/ml) and harvested at different time points. **J** Ubiquitination levels of UHRF1 in cells treated with control or β-TrCP1 siRNAs as indicated. **K** The IHC and correlation analysis showing the reverse associations between SIRT6 and UHRF1 levels in BLCA samples. *p < 0.05, **p < 0.01, ***p < 0.001, ns no significance

To explore the underlying mechanisms downstream of SIRT6, we constructed double-tagged FLAG-HA-SIRT6 plasmids and transfected them into 293 T cells to overexpress SIRT6. Tandem Affinity Purification (TAP) was conducted after the SIRT6-interacting protein complex was enriched and isolated. Next, a series of peptides, such as BAG6, CBWD1, ZNF865, and UHRF1, were identified in the complex (Figure S2A). Considering that UHRF1 is an epigenetic modifier aberrantly overexpressed in many cancers, we intended to focus on the relationships between SIRT6 and UHRF1. To validate this possibility, the co-immunoprecipitation (Co-IP) assay was performed in T24 cells, and endogenous interactions between SIRT6 and UHRF1 were reciprocally confirmed (Fig. 3A, B). Then, we wondered whether SIRT6 deacetylase activity contributes to acetylation levels of UHRF1. Importantly, we observed that the endogenous acetylation of UHRF1, detected by the anti-pan-acetyl K antibodies, was decreased in the presence of SIRT6 overexpression in 253J cells (Fig. 3C). In contrast, the endogenous acetylation of UHRF1 was increased when SIRT6 was depleted, suggesting that SIRT6 mediates the acetylation of UHRF1 (Fig. 3D). Previous studies have reported that K490 is the major acetylation site of UHRF1, and we correspondingly generated the UHRF1-K490R mutant. Indeed, when we transfected UHRF1 (wild-type, WT) or K490R mutant into UHRF1-KD cells, we found that acetylation of UHRF1-K490R was decreased (Fig. 3E). SIRT6 overexpression could only suppress the acetylation of UHRF1-WT, whereas it has a limited effect on the acetylation levels of the UHRF1-K490R mutant (Fig. 3E). Aberrant acetylation may influence the stability of proteins, and we questioned whether SIRT6-modulated deacetylation could mediate the ubiquitination process of UHRF1. Importantly, we showed that SIRT6 knockdown could significantly increase UHRF1 proteins, but not the UHRF1 mRNA levels (Fig. 3F). SIRT6 deficiency could significantly decrease the ubiquitination levels of UHRF1 proteins, compared with those in control cells (Fig. 3G). The wild-type SIRT6, but not the H133Y mutant with deficient deacetylase activity, could effectively prolong the half-time of UHRF1 proteins (Fig. 3H, I). In contrast, SIRT6 overexpression could notably increase the ubiquitination levels of UHRF1 (Fig. 3J). As reported, the E3 ubiquitin ligase β-TrCP1/2 mediated the ubiquitination and degradation of UHRF1 [22]. SIRT6 overexpression mediates the deacetylation of UHRF1 that promotes the ubiquitination of the β-TrCP1/2 on UHRF1 (Fig. 3J). The negative relationship in expression levels of SIRT6 and UHRF1 was further validated in BLCA samples via IHC assays (Fig. 3K). Together, these findings indicate that SIRT6-mediated deacetylation destabilizes UHRF1 proteins.

### SIRT6 deficiency depended on UHRF1 to accelerate BLCA growth and metastasis

**Fig. 4:**
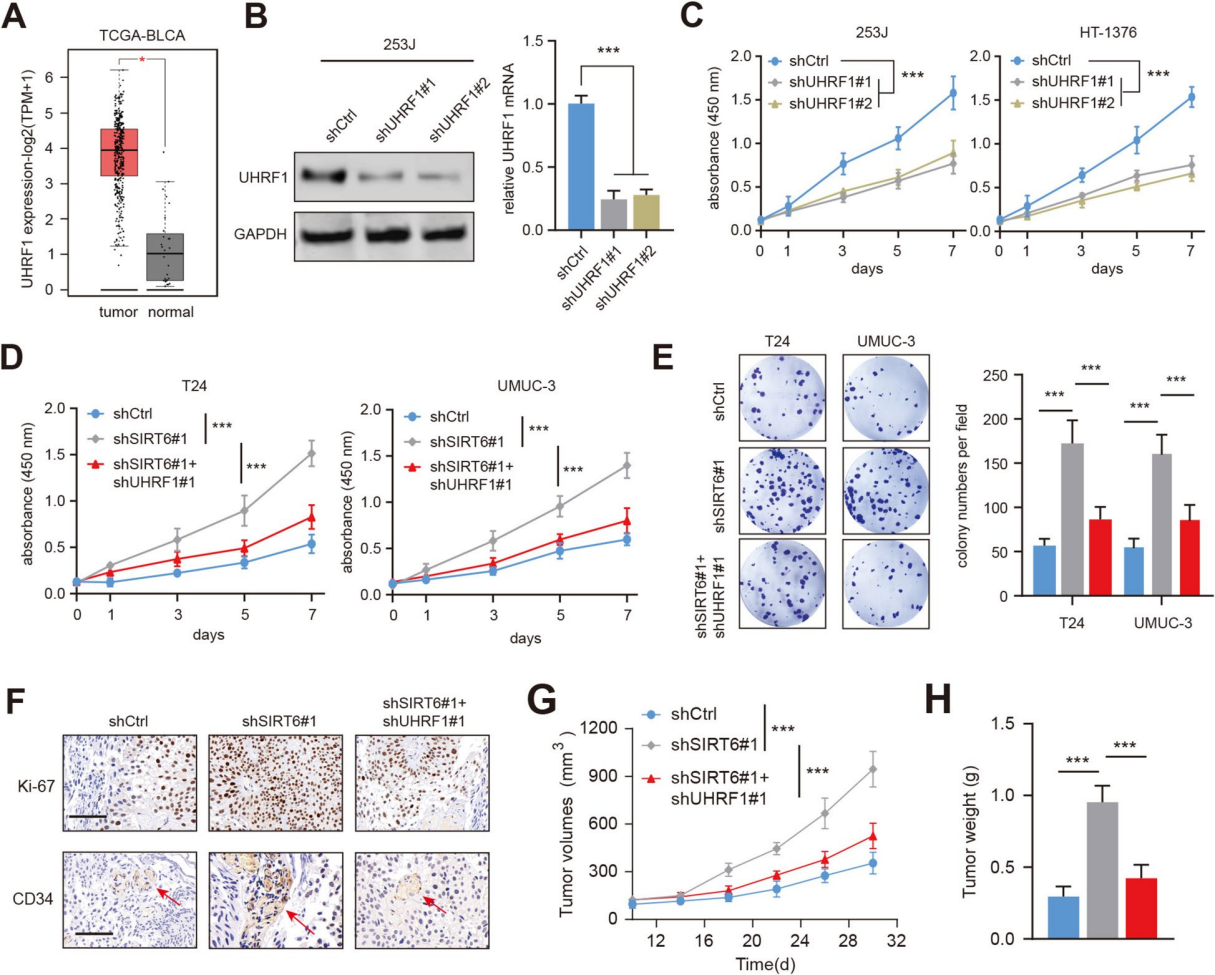
UHRF1 underlies SIRT6-deficiency-mediated BLCA progression.** A** Differential analysis of UHRF1 levels in normal and tumor samples from TCGA-BLCA cohort.** B** 253J cells were transfected with shUHRF1#1 and shScramble, and expressions of UHRF1 were detected with western blotting and RT-qPCR assays. **C** CCK-8 assay showed that cell viability was decreased in 253J and HT-1376 cells. **D-E** CCK-8 assay and colony formation assay revealed that cell viability was decreased in SIRT6-KD T24 and UMUC-3 cells after UHRF1 knockdown. **F** Indicated UMUC-3 cells were subcutaneously injected into BALB/c nude mice. Immunohistochemical detection of Ki-67 and CD34 in tumors derived from indicated cells were shown. **G-H** Quantification of tumor growth curve and tumor weights were compared among indicated tumors. *p < 0.05, **p < 0.01, ***p < 0.001, ns no significance

We thus questioned whether elevated UHRF1 levels contribute to tumorigenesis in SIRT6-deficient BLCA. Then, we analyzed the TCGA-BLCA transcriptome data and found that UHRF1 expressions were aberrantly up-regulated in tumor samples relative to those in normal samples (Fig. 4A). Besides, UHRF1 expressions were also markedly reduced with shRNA#1 and shRNA#2 treatment of 253J cells, as confirmed by RT-qPCR and western blotting assays (Fig. 4B). Indeed, CCK-8 assays suggested that UHRF1 inhibition via shRNAs could notably suppress BLCA cell proliferation rates (Fig. 4C). Furthermore, we knocked down UHRF1 in SIRT6-KD BLCA cells and observed that fast growth efficiency of SIRT6-deficient cells could be abrogated with UHRF1-KD treatment (Fig. 4D). Colony formation assays further validated this finding that UHRF1 inhibition could hinder the growth of colonies formed by SIRT6-deficient cells (Fig. 4E). Additionally, in the wound-healing and transwell experiments, SIRT6 deficiency could accelerate cell migration or invasion, which could be substantially weakened by UHRF1 knockdown in T24 cells (Figure S2B-D). Lastly, we assessed the in vivo functions of SIRT6-UHRF1 axis in BLCA and generated a xnenograft tumor model by subcutaneously injecting UMUC-3 cells, which had been transfected with SIRT6#1 shRNA or control scramble shRNA. As implicated from the xenograft mice model, SIRT6 depletion enhanced tumor growth and angiogenesis, which could be significantly impaired by UHRF1-KD in UMUC-3 cells (Fig. 4F–H). In addition, we overexpressed UHRF1 in SIRT6-overexpreessing 253J and HT-1376 cells. We observed that SIRT6-OE could impair the cell growth and migration capacities, which could be resuced by the ectopic expression of UHRF1 (Figure S2E-F). Together, these assays suggested that the SIRT6-KD-induced phenotypes could be destructed by UHRF1 knockdown in BLCA.

### UHRF1 enhanced SIRT6-deficient BCLA cells glycolysis and lactate secretion

**Fig. 5:**
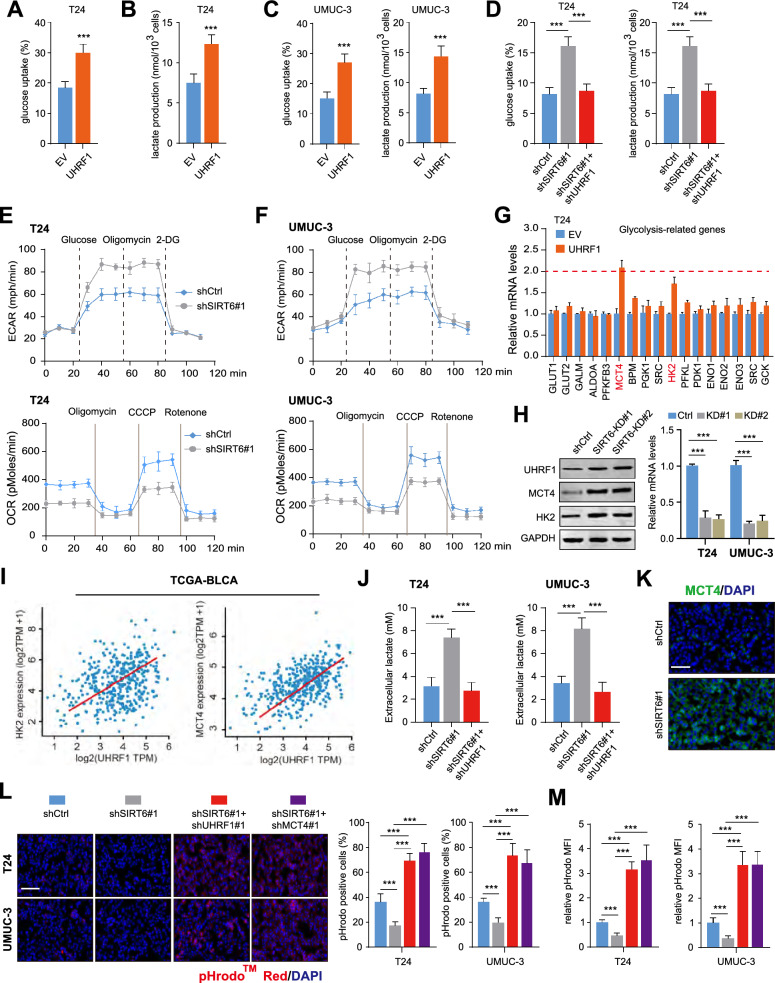
UHRF1 regulates glycolysis and lactate secretion in SIRT6-KD BLCA cells. **A** Overexpression of UHRF1 induced glucose uptake (left) and lactate production (right) in T24 cells. **B** Overexpression of UHRF1 induced glucose uptake (left) and lactate production (right) in UMUC-3 cells. **(C)** UHRF1 overexpression enhanced the glucose uptake and lactate production in T24 cells. **D** UHRF1-KD reduced glucose uptake and lactate production in SIRT6-KD T24 cells. **E–F** The ECAR profile was monitored in SIRT6-KD T24 (**E**) and UMUC-3 (**F**) cells with a Seahorse XF24 analyser for 100 min. The metabolic inhibitors were injected sequentially at different time points as indicated. **G** MCT4 and HK2 were screened as UHRF1-regulated genes. The expressions of a panel of glucose metabolism-related genes were detected by RT-qPCR analysis in UHRF1-overexpressing and control cells. **H** Western blotting and RT-qPCR analysis were used to detect MCT4/HK2 levels in T24 or UMUC-3 cells with or without SIRT6 knockdown. **I** Correlation analysis was calculated to determine UHRF1 and MCT4/HK2 expressions in TCGA-BLCA cohort. **J** Extracellular lactate levels of SIRT6 deficient cell lines with or without UHRF1-KD (n = 3). **K** Immunofluorescence staining of MCT4 in control SIRT6-KD tumors. Bar, 100 μm. (n = 3). **L-M** Intracellular pH was detected by pHrodo Red, and representative images were shown in indicated cells with or without UHRF1/MCT4-KD. Bar, 100 μm. (n = 3). *p < 0.05, **p < 0.01, ***p < 0.001, ns no significance

To thoroughly investigate how UHRF1 regulates BLCA progression and metastasis, we obtained the expression matrix of TCGA-BLCA samples and divided them into UHRF1-high and UHRF1-low groups. Gene Set Enrichment Analysis (GSEA) indicated the positive relationships between UHRF1 and glycolysis in BLCA (Figure S2G). Therefore, we considered whether UHRF1 manipulated glycolysis to potentiate BLCA malignant phenotypes. To address this issue, we detected the glycolytic activities in UHRF1-overexpressing cells. As expected, up-regulation of UHRF1 could notably increase glucose uptake and lactate production in BLCA cells (Fig. 5A–C). Considering that SIRT6 negatively modulates UHRF1 expressions, we found that SIRT6 deficiency could also strengthen glucose uptake and lactate production, which could be completely impaired by UHRF1-KD (Fig. 5D). To further confirm the role of UHRF1 in modulating aerobic glycolysis in BLCA, we carried out the glycolytic measurement with Seahorse Extracellular Flux Analyser. As suggested by the extracellular acidification rate (ECAR) kinetic profiles, we found SIRT6 deficiency significantly inhibited the glycolytic activity in T24 and UMUC-3 cells (Fig. 5E). Moreover, when SIRT6 was inhibited, the OCR decreased accordingly in T24 and UMUC-3 cells, supporting its negative role in mitochondrial respiration (Fig. 5F). To further demonstrate the underlying mechanisms by which UHRF1 regulates in BLCA, we detected the transcription of a panel of glucose metabolism-associated signature in UHRF1-overexpressing cells and explore whether these genes were potentially modulated by UHRF1. Intriguingly, we found that only expressions of MCT4 and HK2 were primarily increased (fold change > 1.5) in response to UHRF1 overexpression (Fig. 5G). Accordingly, SIRT6-KD could significantly increase the expressions of UHRF1/MCT4/HK2 in T24 and UMUC-3 cells, as indicated by RT-qPCR and western blotting assays (Fig. 5H). Positive relationships between UHRF1 and MCT4/HK2 expressions were further demonstrated in samples from TCGA-BLCA cohort (Fig. 5I). CCK-8 assays confirmed that SIRT6 deficiency relied on MCT4/HK2 to enhance BLCA cell growth (Figure S2H). MCT4, encoded by SLC16A3, is responsible for lactate transportation and mainly involved in maintaining intracellular pH homeostasis in tumor microenviroment. We thus assessed the impact of SIRT6 deficiency on lactate production in BLCA tumor cells. First, we found that SIRT6-deficient tumor cells produced higher extracellular lactate levels than their control cells, which could be abolished with UHRF1-KD (Fig. 5J). We further confirmed a significantly increased level of MCT4 in SIRT6-deficient BLCA tumors, as shown by immunofluorescence staining assays (Fig. 5K). We then assessed the intracellular pH following SIRT6-KD through immunofluorescence and flow cytometry methods, and found that the intracellular pH was significantly higher caused by SIRT6 deficiency (Fig. 5L). UHRF1 or MCT4 inhibition significantly decreased the intracellular pH in SIRT6-KD cells (Fig. 5M). Taken together, our data suggested that SIRT6 deficiency promoted the glycolysis activity and enhanced lactate secretion via activating UHRF1/MCT4.

### UHRF1 inhibitor (NSC232003) is effective to inhibit SIRT6-low BLCA progression

**Fig. 6:**
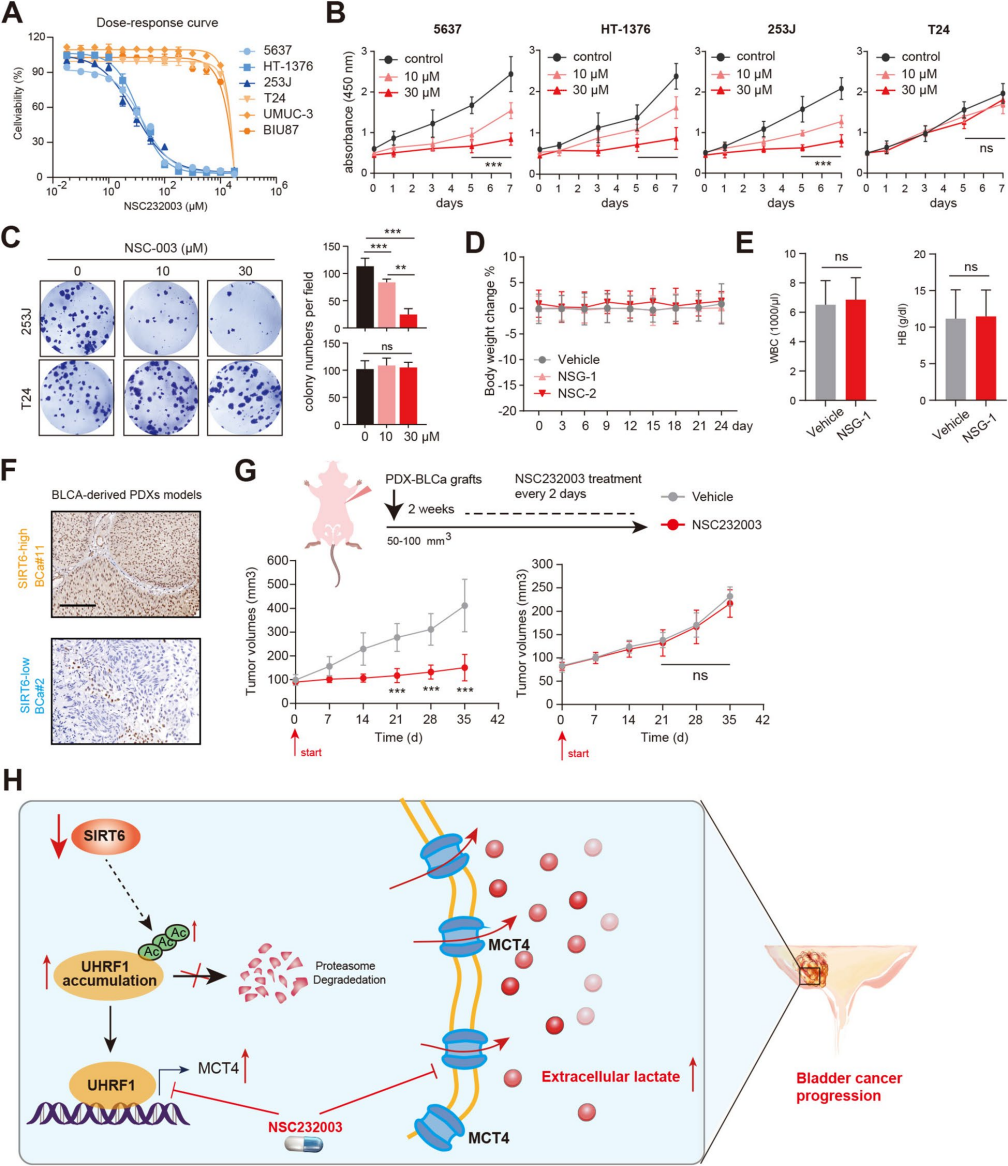
UHRF1 inhibitor (NSC232003) specifically inhibits SIRT6-deficient BLCA. **A** Dose–response curves and IC50 in a panel of BLCA cells treated with NSC232003. Data are presented as mean + / − SD (n = 6) from one-of-three independent experiments. **B** CCK-8 analysis showed the cell viability of indicated cells treated with increasing doses of NSC232003. **C** Colony formation assays showed the sensitivity of indicated cells to increasing doses of NSC232003. **D-E** Mouse weight changes measurements **D** (n = 5) and complete blood counts **E** assessed on vehicle control and NSC232003-treated mice. **F** Representative immunohistochemical staining against SIRT6 in PDXs derived from SIRT6-low and -high BLCA specimens. **G** Tumor volumes of indicated PDXs treated with NSC232003 (n = 6). **H** Illustration of SIRT6-UHRF1-MCT4 axis in regulating BLCA lactate secretion and progression. *p < 0.05, **p < 0.01, ***p < 0.001, ns no significance

Given that UHRF1 is required for SIRT6-deficient BLCA in driving tumor growth, we therefore questioned whether the UHRF1 specific inhibitor, NSC232003, could effectively suppress BLCA. To address this point, we obtained the SIRT6-high (T-24, UMUC-3, BIU87), and SIRT6-low BLCA (5637, HT-1376, 253J) cell lines. Intriguingly, we found that SIRT6-low cell lines were more sensitive to NSC232003 with half-maximal inhibitory concentrations (IC50) < 100 μM (Fig. 6A). Nevertheless, SIRT6-high cell lines showed notable resistance to NSC232003, of which the half-maximal inhibitory concentrations (IC50) were more than 10,000 μM (Fig. 6A). Consistently, CCK-8 assays further implicated that NSC232003 could inhibit the growth rates of SIRT6-low cell lines in a dose-dependent manner (Fig. 6B). However, T24 cells were irresponsive to NSC232003 treatment with the above concentrations (Fig. 6B). Treatment with NSC232003 further led to potent inhibition of colony formation capacities in 253J cells, whereas inducing negligible effects on T24 cells (Fig. 6C).

Furthermore, we pharmacologically characterised NSC232003 in murine BLCA models, and detected the in vivo toxicological effects of NSC232003. Notably, NSC232003 treatments showed no evident toxicity in immuno-competent mice, and did not disturb the body weight, blood cell counts, or organ morphology (Fig. 6D, E, Figure S3A). To confirm the clinical significance of NSC232003, we obtained SIRT6-low and SIRT6-high BLCA samples to construct the patient-derived xenograft (PDX) models that show distinct SIRT6 expressions (Fig. 6F). In line with our expectations, NSC232003 treatment was more efficient in suppressing SIRT6-low PDX tumors than those derived from SIRT6-high PDXs, as quantified by tumor growth curves (Fig. 6G). Taken together, we implicate that SIRT6-low BLCA could benefit from NSC232003 treatment.

## Discussion

Intensive studies have shown that abnormal epigenetic events may contribute to metabolic disorders in multiple tumors [23]. The aberrant interplay between epigenetic modifications and metabolic reprogramming enhanced BLCA tumorigenesis, immune evasion, and drug resistance [24]. In this study, we focused on the novel role of human deacetylase SIRT6 in BLCA. Bioinformatic analysis revealed that SIRT6 was down-regulated in MIBC and correlated with poor prognosis of patients. SIRT6 inhibition significantly enhanced colony formation, self-renewal, and migration abilities. SIRT6 deficiency further promoted BLCA metastasis in vitro and in vivo. Mechanistically, SIRT6 interacts with UHRF1 and deacetylates UHRF1. Then, SIRT6 promotes β-TrCP1-mediated UHRF1 degradation, thus SIRT6-mediated deacetylation destabilizes UHRF1. Functional experiments suggested that down-regulated SIRT6 depended on UHRF1 to accelerate BLCA cell proliferation, migration, and in vivo growth. Additionally, UHRF1 was shown to positively correlate with the glycolysis pathway, and UHRF1 up-regulation in BLCA cells notably increased glucose uptake and lactate production. UHRF1 transcriptionally activates MCT4 and HK2 to induce a substantial increase in glycolytic activity. SIRT6 deficiency depended on UHRF1 to drive MCT4-dependent lactate secretion in BLCA. Lastly, we demonstrated that UHRF1 inhibitor NSC232003 could specifically inhibit SIRT6-low/deficient BLCA progression.

The epigenetic reader UHRF1 is found to be an oncogene that is overexpressed in multiple human cancer cells [25]. Five functional domains were found in UHRF1 proteins, including the UBL (ubiquitin-like) domain, TTD (Tandem Tudor Domain), PHD (Plant Homeo Domain) domain, SRA (Set and Ring Associated) domain, and RING (Really Interesting New Gene) domain [26, 27]. UHRF1 relies on these domains to interact with multiple proteins and constitute a large epigenetic remodeling machinery. For instance, UHRF1 utilizes the SRA domain to interact with DNMT1, which is required for the maintenance of DNA methylation [28]. The TTD domain shows preferential affinity for methylated histones and therefore links DNA methylation to histone modifications. Recent studies have indicated that UHRF1 plays essential role in the development of cancer cells. UHRF1 drives aerobic glycolysis and proliferation via inhibiting SIRT4 in pancreatic cancer [29]. Besides, UHRF1 directly interacts with SAP30 to repress MXD4, which is required for maintaining self-renewal of stem/progenitor cells [30]. In colon cancer, UHRF1 histone- and hemimethylated DNA binding functions epigenetically suppressed the levels of tumor suppressor genes (TSGs) and governed the maintenance of DNA methylation patterns [31]. Intriguingly, UHRF1 also belongs to an E3 ubiquitin ligase, and UHRF1 acetylation substantially enhances its E3 ligase activity [27]. As reported, UHRF1-mediated ubiquitination regulates EZH2 proteins to induce differentiation phenotypes in melanoma [32]. Therefore, UHRF1 possesses the dual functions of chromatin regulation and ubiquitination. Here, we found that UHRF1 activates the expression levels of MCT4 and HK2 via the transcriptional regulations. Previous studies also confirmed the positive regulations between UHRF1 and glycolysis, in which UHRF1 epigenetically suppressed SIRT4 that inhibited the HIF1a/glycolysis axis [29]. In line with the results, UHRF1 could directly activate the glycolytic process by inducing MCT4/HK expressions, providing a novel UHRF1-driven crosstalk in glycolytic reprogramming. Furthermore, Hao Chen et al. found that the proteasomal degradation of UHRF1 is mediated by the SCF(β-TrCP) E3 ligase, and its phosphorylation is required for the recognition of UHRF1 by β-TrCP [22]. In this study, we implicated that UHRF1 deacetylation by SIRT6 was also indispensable for β-TrCP1-mediated ubiquitination. Similarly, another protein, deacetylase, SIRT7, deacetylates and promotes SMAD4 degradation mediated by β-TrCP1, and SIRT7 loss stimulates TGF-β signaling to potentiate epithelial-to-mesenchymal transition [33]. As a result, we uncovered novel upstream mechanisms that determine UHRF1 stability.

MCT4, encoded by solute carrier family 16 member 3 (SLC16A3), is mainly induced by the hypoxia/HIF axis and is responsible for lactic acid transportation across the plasma membrane [34]. Previous studies have shown that MCT4 is overexpressed in various tumors, including hepatocellular carcinoma, lung adenocarcinoma, and breast cancer [35–37]. MCT4-mediated lactate secretion is indispensable for tumor progression. For instance, LKB1 deficiency leads to enhanced lactate production and secretion through up-regulating the MCT4 transporter [36]. Suppressing MCT4-mediated lactate secretion attenuates M2 macrophage polarization in LKB1-deficient tumor cells. In this study, SIRT6 deficiency activates the UHRF1/MCT4 axis to increase glycolytic and oxidative phosphorylation (OXPHOS) rates, as assessed by ECAR and OCR quantification. SIRT6 loss depended on UHRF1 to enhance cell viability, ATP production, and extracellular lactate accumulation. Targeting UHRF1/MCT4 could notably abrogate the lactate export and cell viability. Therefore, we at least explained the reasons that the SIRT6/UHRF1 axis contributes to the regulations of MCT4-mediated lactate secretion in BLCA.

However, some limitations in this study need to be improved. First of all, due to limited BLCA samples in this project, we did not provide an appropriate cutoff to divide the SIRT6^high^ and SIRT6^low^ BLCA groups. In the future, our team will enroll more suitable BLCA patients from multi-centers to conduct IHC assays. Besides, we did not discuss the relationships between SIRT6/UHRF1/MCT4 axis and antitumor immunity in BLCA. Since previous work indicated that lactate could strengthen anti-tumor immunity by increasing CD8^+^ T cells in multiple tumor models [38, 39], more immunological assays were warranted to confirm the associations between SIRT6/UHRF1 and immune evasion in BLCA. In addition, we should generate more BLCA models, like the orthotopic bladder cancer model and BLCA patient-derived organoids (PDOs), to thoroughly assess the pharmacological effects of NSC232003. Last of all, UHRF1 is shown to be an essential TF in transcriptional regulation. Therefore, NSC-003 does not specifically target MCT4/HK2 gene expression. We should explore other UHRF1-regulated targets that may participate in BLCA progression.

In conclusion, our work uncovered the essential role of the SIRT6-UHRF1-MCT4 axis in modulating BLCA glycolytic reprogramming and lactate secretion (Fig. 6H). Targeting UHRF1 with a specific inhibitor, NSC232003 has clinical significance in inhibiting SIRT6-deficient BLCA progression.

## Supplementary Information

Supplementary material 1.

## Data Availability

The data used to support the findings of this study are available from the corresponding author upon request.
