# Supplementary material for: SIRT6 deficiency impairs the deacetylation and ubiquitination of UHRF1 to strengthen glycolysis and lactate secretion in bladder cancer
Source: Cell Biosci. 2024 Dec 21;14:153. doi: 10.1186/s13578-024-01333-2 (PMC11663349; doi:10.1186/s13578-024-01333-2)
Supplement: Supplementary file 1 — Supplementary material 1. [file 13578_2024_1333_MOESM1_ESM.docx]

**Supplementary Figures and legends**

**
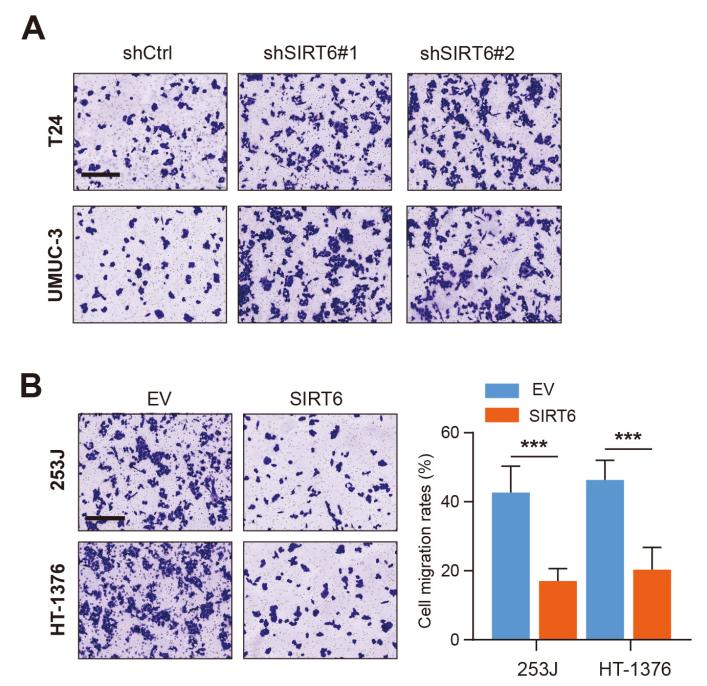
**

**Figure S1 SIRT6 regulates BCLA migration abilities in vitro. (A)** Representative graphs of Transwell assays for control and SIRT6-KD T24 and UMUC-3 cells. **(B)** Representative graphs of Transwell assays for control and SIRT6-OE 253J and HT-1376 cells.

**
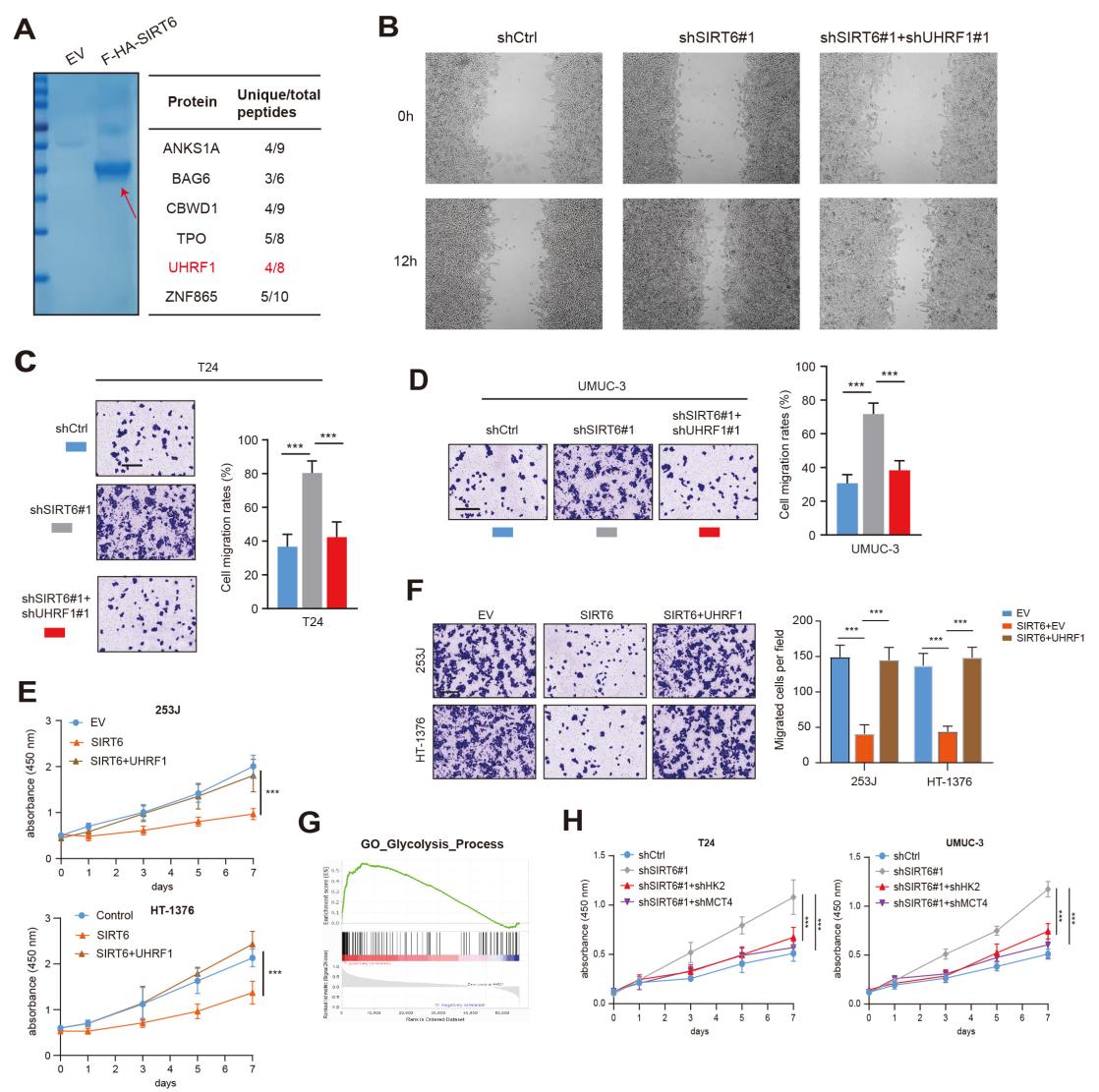
**

**Figure S2. SIRT6-UHRF1 regulating axis in BLCA progression. (A)** A vector or FLAG-HA-SIRT6 plasmid was transfected into 293 T cells. After treatment with 10 μM MG132 for 4 h, cell lysate was immunoprecipitated with anti-FLAG and anti-HA beads, and the proteins that interact with SIRT6 were enriched. A Coomassie gel graph was shown in the left panel and representative proteins were selected to be shown in the right panel. **(B)** Representative graphs of wound-healing assays for SIRT6-KD T24 cells after UHRF1 knockdown are shown at 0 and 12 h. **(C-D)** Representative graphs of transwell assays for control and SIRT6-KD T24 and UMUC-3 cells with or without UHRF1-KD. **(E)** CCK-8 assays showing the cell growth in SIRT6-OE 253J or HT-1376 cells with or without ectopic expression of UHRF1. **(F)** Transwell assays showing the cell migration abilities in SIRT6-OE 253J or HT-1376 cells with or without ectopic expression of UHRF1. **(G)** GSEA analysis was performed between UHRF1-high and UHRF1-low samples. **(H)** CCK-8 assays showing the cell growth in SIRT6-KD T24 or UMUC-3 cells with or without HK2/MCT4-KD.

**
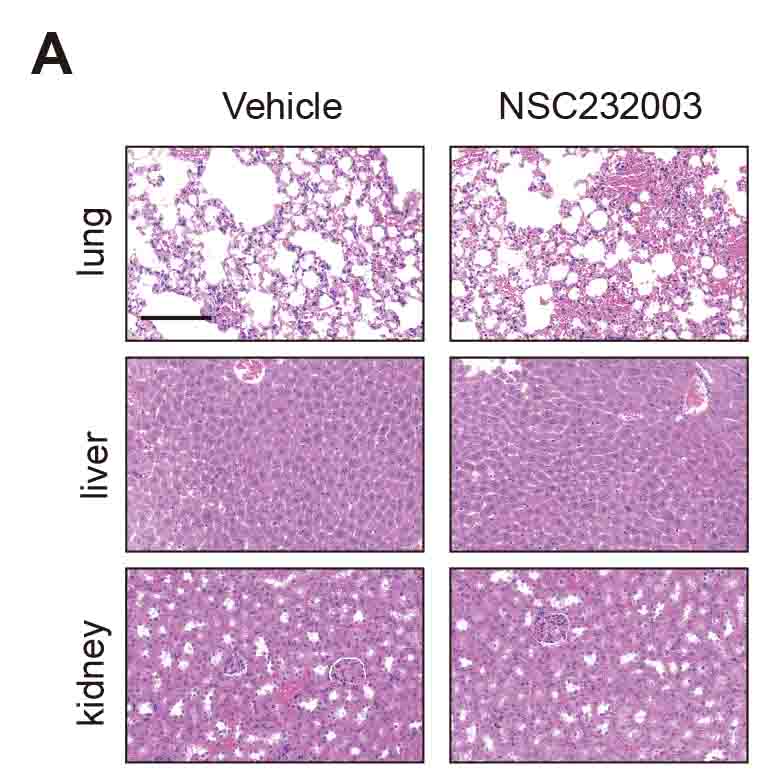
**

**Figure S3. Assessment of toxicology of NSC232003 in vivo. (A)** Representative HE pictures revealing the morphology of critical organs in mice treated with NSC232003.
